# Mechanisms of Fatal Cardiotoxicity following High-Dose Cyclophosphamide Therapy and a Method for Its Prevention

**DOI:** 10.1371/journal.pone.0131394

**Published:** 2015-06-26

**Authors:** Takuro Nishikawa, Emiko Miyahara, Koichiro Kurauchi, Erika Watanabe, Kazuro Ikawa, Kousuke Asaba, Takayuki Tanabe, Yasuhiro Okamoto, Yoshifumi Kawano

**Affiliations:** 1 Department of Pediatrics, Graduate School of Medical and Dental Sciences, Kagoshima University, Kagoshima, Japan; 2 Department of Clinical Pharmacy and Pharmacology, Graduate School of Medical and Dental Sciences, Kagoshima University, Kagoshima, Japan; 3 Department of Clinical Pharmacotherapy, Hiroshima University, Hiroshima, Japan; 4 Clinical Development Dept. 1, Clinical Development Division, EPS Corporation, Saitama, Japan; University of Central Florida, UNITED STATES

## Abstract

Observed only after administration of high doses, cardiotoxicity is the dose-limiting effect of cyclophosphamide (CY). We investigated the poorly understood cardiotoxic mechanisms of high-dose CY. A rat cardiac myocardial cell line, H9c2, was exposed to CY metabolized by S9 fraction of rat liver homogenate mixed with co-factors (CYS9). Cytotoxicity was then evaluated by 3-(4,5-dimethyl-2-thiazolyl)¬2,5-diphenyl¬2*H*-tetrazolium bromide (MTT) assay, lactate dehydrogenase release, production of reactive oxygen species (ROS), and incidence of apoptosis. We also investigated how the myocardial cellular effects of CYS9 were modified by acrolein scavenger N-acetylcysteine (NAC), antioxidant isorhamnetin (ISO), and CYP inhibitor β-ionone (BIO). Quantifying CY and CY metabolites by means of liquid chromatography coupled with electrospray tandem mass spectrometry, we assayed culture supernatants of CYS9 with and without candidate cardioprotectant agents. Assay results for MTT showed that treatment with CY (125–500 μM) did not induce cytotoxicity. CYS9, however, exhibited myocardial cytotoxicity when CY concentration was 250 μM or more. After 250 μM of CY was metabolized in S9 mix for 2 h, the concentration of CY was 73.6 ± 8.0 μM, 4-hydroxy-cyclophosphamide (HCY) 17.6 ± 4.3, *o*-carboxyethyl-phosphoramide (CEPM) 26.6 ± 5.3 μM, and acrolein 26.7 ± 2.5 μM. Inhibition of CYS9-induced cytotoxicity occurred with NAC, ISO, and BIO. When treated with ISO or BIO, metabolism of CY was significantly inhibited. Pre-treatment with NAC, however, did not inhibit the metabolism of CY: compared to control samples, we observed no difference in HCY, a significant increase of CEPM, and a significant decrease of acrolein. Furthermore, NAC pre-treatment did not affect intracellular amounts of ROS produced by CYS9. Since acrolein seems to be heavily implicated in the onset of cardiotoxicity, any competitive metabolic processing of CY that reduces its transformation to acrolein is likely to be an important mechanism for preventing cardiotoxicity.

## Introduction

Cyclophosphamide (CY) is an alkylating agent that has both potent immunosuppressive properties and antineoplastic activity. Consequently, high-dose CY is a mainstay in most conditioning regimens for hematopoietic stem cell transplantation (HSCT) [1, 2]. Recently, administration of posttransplantation CY (PTCy) in high doses has been attracting attention as novel strategy for preventing graft-versus-host disease (GVHD). High-dose CY, when administered at the appropriate time after transplantation, depletes alloreactive T cells from the donor and host and can inhibit both GVHD and graft rejection [3, 4].

**Fig 1:**
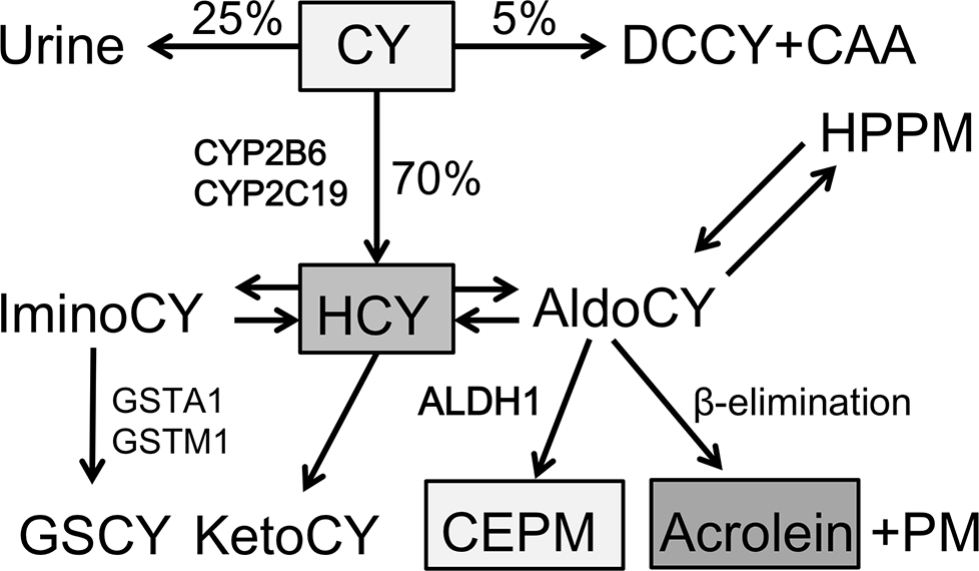
Cyclophosphamide metabolic pathways. Cyclophosphamide (CY) is metabolized to 4-hydroxy-cyclophosphamide (HCY) in the hepatic cytochrome P-450 enzyme (CYP) system (CYP2B6 and/or CYP2C19). HCY enters cells as tautomer aldocyclophosphamide (AldoCY). Through β-elimination, AldoCY can be converted to phosphoramide mustard (PM) and acrolein. Alternatively, AldoCY can also be oxidized to the inactive metabolite *o*-carboxyethylphosphoramide mustard (CEPM) by aldehyde dehydrogenase 1 (ALDH1). Other metabolites include chloroacetaldehyde (CAA), deschloroethyl-cyclophosphamide (DCCY), 4-keto-cyclophosphamide (KetoCY), hydroxypropyl-phosphoramide mustard (HPPM), imino-cyclophosphamide (IminoCY), and glutathionyl-cyclophosphamide (GSCY).

CY is activated by the hepatic cytochrome P-450 (CYP) enzyme system to form 4-hydroxy-cyclophosphamide (HCY), which is in equilibrium with aldocyclophosphamide (AldoCY). Depending on the type of cell, AldoCY may, through the chemical process of β-elimination, decompose to form cytotoxic phosphoramide mustard (PM) and byproduct acrolein, or may be oxidized by aldehyde dehydrogenase 1 (ALDH1) to the inactive metabolite *o*-carboxyethylphosphoramide mustard (CEPM) (Fig 1) [5, 6].

The dose-limiting toxic effect of CY, observed only after administration of high-doses, is cardiotoxicity. Since its initial description 40 years ago [1], the wide-spectrum incidence, manifestation, and severity of cardiotoxicity has been widely reported [7–9]. Most severely, with attendant risk of sudden death, this may manifest as hemorrhagic necrotic perimyocarditis. On the basis of postmortem examination, the pathophysiology of high-dose CY-associated cardiac toxicity is thought to depend upon toxic endothelial damage followed by extravasation of toxic metabolites with resultant myocyte damage and interstitial hemorrhage and edema [7, 10]. Since the mechanism underlying this phenomenon has not yet been elucidated, and no definitive risk factors have yet been identified, we investigated the cardiotoxic mechanisms of high-dose CY. To determine preliminary mechanisms that may prevent the occurrence of CY-induced cytotoxicity in H9c2 embryonic rat cardiomyocytes cell line, we also evaluated the protective effects of potential cardioprotectant agents (PCA).

## Materials and Methods

### Reagents

CY, dimethyl sulfoxide (DMSO), N-acetylcysteine (NAC), and isorhamnetin (ISO) were purchased from SIGMA-Aldrich (St. Louis, MO), HCY and CEPM were purchased from Santa Cruz Biotechnology (Dallas, TX). Acrolein was purchased from AccuStandard (New Haven, CT). β-ionone (BIO) was purchased from Wako (Osaka, Japan).

### Measurement of CY, HCY and CEPM concentration in plasma from patients treated with high-dose CY

To determine the concentration of CY in this experiment, we measured CY and CY metabolites in blood plasma samples from patients treated with high-dose CY (60 mg/kg) administered in a 2-hour constant infusion. Samples of 2 mL of venous blood were collected before the start of CY and at 3, 7, 9, 20 and 24 hours on day 1 and day 2 of CY treatment. The blood samples were centrifuged immediately at 2,000 rpm for 15 minutes and the plasma stored at –80°C until analysis. To evaluate possible myocardial damage, each patient underwent a standard 12-lead electrocardiogram (ECG) before every administration of high-dose CY.

Analysis of CY and CEPM was conducted according to the following methods. After solid-phase extraction with Sep-Pak C18 3mL Vac cartridge (Waters, Milford, MA), each plasma sample was analyzed on a C18 column (3.5 μm; 100 mm × 2.1 mm i.d.: Xbridge BEH, Waters, Milford, MA) with liquid chromatography/tandem mass spectrometry (LC/MS/MS) using a liquid chromatograph coupled to a ABsciex Triple Quad 4500. Positive electrospray ionization was employed as the ionization source. After reconstitution to mobile phase with acetonitrile/0.066% ammonia solution (7:3, v/v), detection was performed by electrospray positive ionization mass spectrometry, monitoring multiple reactions for transitions of CY at m/z 260.98 to 140.00 and of CEPM at m/z 292.96 to 220.90.

For determination of HCY, plasma was derivatized with hexamethylphosphoramide and acetonitrile/methanol (1:1, v/v) was added to the derivative. After centrifuging the samples, the organic layer was removed and dried with a gentle stream of nitrogen at 50°C. The sample was reconstituted to mobile phase and then analyzed, as above, on a C18 column interfaced with a triple quadruple tandem mass spectrometer using electrospray ionization, monitoring multiple reactions for transitions of HCY at m/z 334.07 to 220.90.

### Cell culture

H9c2 embryonic rat cardiomyocytes cell line and HL-60 human promyelocytic leukemia cell line specimens were purchased from American Type Culture Collection (ATCC: Manassas, VA). The H9c2 specimens were maintained in DMEM medium (Life Technologies, Tokyo, Japan) containing 10% fetal bovine serum (FBS) (ATCC) at 37°C in a humidified atmosphere with 5% carbon dioxide (CO_2_). HL-60 specimens were maintained in RPMI-1640 medium (SIGMA-Aldrich) containing 10% FBS at 37°C in a humidified atmosphere with 5% CO_2_.

### Analysis of cell viability

H9c2 cells were plated at 2.5 × 10^4^ cells/mL density in 24-well plates and grown overnight. These samples were exposed to CY (125–500 μM) at 37°C in 5% CO_2_ for 24 or 48 hours. To metabolize CY, we added 83.3 μL S9 fraction (16.7%) of rat-liver homogenate mixed with co-factors to each sample (S9 mix; Oriental Yeast Co, Ltd., Tokyo, Japan) to H9c2 cell culture samples (CYS9). After 2 hours, to remove the CY, CY metabolites and S9 mix, the media were replaced with fresh DMEM medium. After adding 50 μL of 5 mg/mL of 3-(4,5-dimethyl-2-thiazolyl)-2,5-diphenyl-2*H*-tetrazolium bromide (MTT: Dojindo, Kumamoto, Japan) to each sample, it is incubated at 37°C, 5% CO_2_ for 2 hours. Formazan crystals were dissolved by adding 1 mL of DMSO and the absorbance was measured at 570 nm, and the background absorbance at 630 nm using an Infinite M200 (Tecan, Mannedorf, Switzerland) and analyzed data using i-Control version 1.0 software (Tecan).

**Fig 2:**
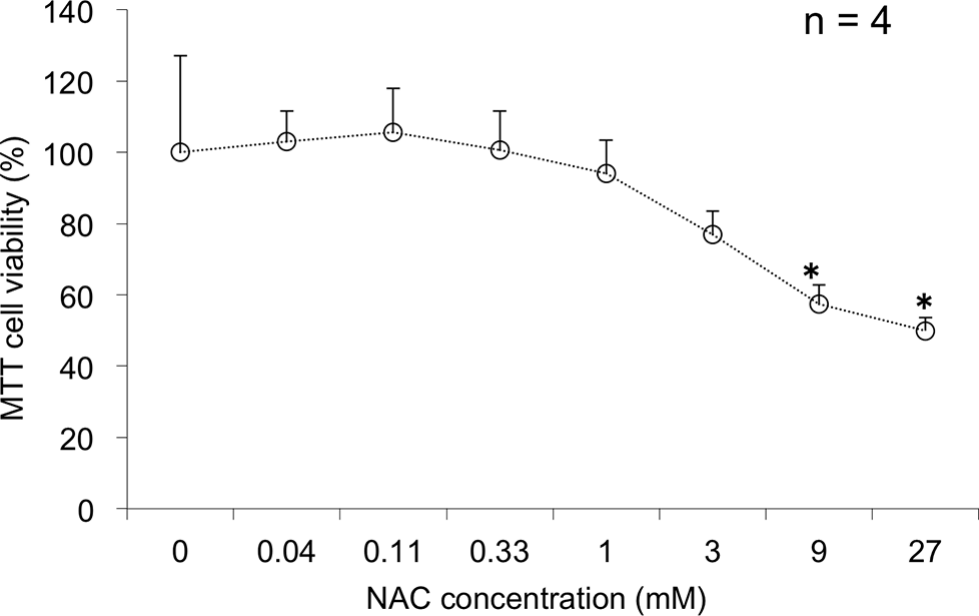
Cytotoxicity of N-acetylcysteine on H9c2 cells. H9c2 cell viability after 24-hour exposure to N-acetylcysteine (NAC) was assessed by MTT assay (mean + standard deviation (SD) from 4 independent experiments). *p < 0.05 compared with control.

To confirm the sublethal concentration of NAC related to its ability to protect cells from CYS9 induced toxicity, we first exposed H9c2 cells with NAC at 0.04 mM, 0.11 mM, 0.33 mM, 1 mM, 3 mM, 9 mM, 27 mM for 24 hours. Untreated cells were used for control. NAC induced significant cytotoxicity at concentrations ≥ 9 mM. Cell viability was further reduced by 60% at 9 mM of NAC (*p* < 0.05) (Fig 2). At 3 mM of NAC, the cell viability did not significantly differ from the control group. We conservatively selected a nontoxic concentration, 1 mM, for antioxidants for the subsequent experiments. Appropriate treatments with isorhamnetin (ISO) and β-ionone (BIO) were based on reports [11, 12].

To determine how well cardiomyocytes were protected, before exposure to CYS9, H9c2 cells were incubated for 2 hours with 10% FBS supplemented DMEM containing 1 mM NAC or for 12 hours containing 25 μg/mL ISO. After pre-treatment, the medium of each sample was replaced with fresh medium containing NAC or ISO and CYS9 was also added. Extended pre-treatment was not required for BIO: 1 μM BIO was added to samples, directly followed by CYS9.

In parallel protocols, the morphology of H9c2 cells after exposure to CYS9 in the 24-well culture plates was observed by microscopy (Axio observer. Z1; Zeiss) at 100× magnification.

To examine the effects of acrolein, H9c2 cells, suspended in 24-well plates at 2.5 × 10^4^/mL density in DMEM with 5% FBS, were incubated overnight and then exposed to different concentrations (10, 30, 100 μM) of acrolein with or without NAC at 37°C for 24 hours or 48 hours. For control, unexposed H9c2 cells were seeded in 24-well plates at 2.5 × 10^4^/mL density in DMEM with 5% FBS and incubated at 37°C in 5% CO_2._ For experiments with NAC, the cells were pre-treated as described above, and then exposed for 24 hours to acrolein at 37°C in 5% CO_2_ with the reagents. After exposure, the viability of the cells was measured using an MTT assay, as described above.

### Measurement of lactate dehydrogenase release

Lactate dehydrogenase (LDH) release, as a marker of cellular injury, was measured in cell culture supernatant. H9c2 cells (1.25 × 10^4^ cells/well) were cultured overnight in 96-well plates. After incubation, the cells were exposed to CY, S9, PCA, and CYS9 with and without PCA at 37°C in 5% CO_2_. For experiments with NAC or ISO, the cells were pre-treated as described above, and then exposed for 2 hours to CYS9 at 37°C in 5% CO_2_ with the respective reagents. For experiments with BIO, the cells were co-incubated with CYS9 plus BIO as described above. After 2 hours exposure, the culture supernatant was removed by replacing sample media with fresh medium. Then after further six hours of incubation, LDH release in the medium of each sample was measured, according to the manufacturer’s instructions, using LDH Cytotoxicity Detection Kits (Takara, Shiga, Japan).

### Measurement of intracellular ROS generation

To detect intracellular superoxide (O_2_•−) and H_2_O_2_, we used dichlorofluorescin diacetate (DCFH-DA) molecular probes purchased from SIGMA-Aldrich. Briefly, in 96-well black flat bottom plates, H9c2 cells were plated at 2.5 × 10^4^ cells/mL density in 10% FBS supplemented DMEM and then, for 1 hour at 37°C in 5% CO_2_, exposed to 250 μM CY or CYS9 (250 μM CY). After exposure, culture supernatants were removed and cells were washed with PBS (phosphate buffer solution). Cells were then incubated with 10 μM of DCFH-DA diluted with PBS. Pre-treatment with NAC, described above in “Analysis of cell viability”, was carried out. After pre-treatment, the cells were exposed to CYS9 for 1 hour at 37°C. Then the supernatants were removed, the cells washed with PBS and finally, as above, incubated with 10 μM of DCFH-DA diluted with PBS.

For selective detection of hypochlorous acid (HOCl) and the hydroxyl radical (•OH), for 1 hour at 37°C, with and without 1 mM NAC, H9c2 cells plated at 2.5 × 10^4^ cells/mL density in 96-well black flat bottom plate were exposed to CY or CYS9 and then incubated with 10 μM aminophenyl fluorescein (APF: Sekisui Medical, Tokyo, Japan) or 10 μM hydroxyphenyl fluorescein (HPF: Sekisui Medical) [13]. For experiments with NAC, cells were pre-treated and exposed as described in the previous paragraph. The specificity and usefulness of these probes has been previously described [14, 15]. We measured the fluorescence intensity of cells using an Infinite M200 (Tecan) and analyzed data using i-Control version 1.0 software (Tecan).

### Measurement of CY, HCY, CEPM and acrolein concentration in cell culture by LC-MS/MS and high performance liquid chromatography

As described above in ‘Analysis of cell viability’, H9c2 cells were exposed to CYS9 for 2 hours. After 1 hour and 2 hours exposure, cell culture supernatants were collected and, as described above, LC-MS/MS was carried out to analyze concentrations of CY, HCY and CEPM. Acrolein, however, was measured using a colorimetric method based on the specific reaction of acrolein with *m*-aminophenol in the presence of hydroxylamine [16]. The reaction mixture (0.2 mL) containing 0.1 mL of plasma, 23 mM *m*-aminophenol, 43 mM hydroxylamine hydrochloride, and 1.5 M HCl was boiled for 10 min. The amount of acrolein was determined by high performance liquid chromatography (HPLC) according to the method of Bohnenstengel et al. [17], using 0.08 mL samples of supernatant obtained after centrifugation. Fluorescence of 7-hydroxyquinoline (acrolein derivative) was measured at an excitation wavelength of 358 nm and an emission wavelength of 510 nm.

### Detection of apoptosis by evaluation of caspase-3 and caspase-7 activity

To detect apoptosis, caspase-3 and caspase-7 activity was evaluated. As described above in ‘Analysis of cell viability’, H9c2 cells were plated at 2.5 × 10^4^ cells/mL density in 24-well plates and exposed to CYS9 for 2 hours. After different treatments, the supernatants were removed and cells were washed with PBS. Then the PBS was replaced with 500 μL of phenol-red-free DMEM supplemented with 10% FBS. Finally, CellEvent Caspase 3/7 Green ReadyProbes reagent (Life Technologies, Carlsbad, CA) was added to each well and incubated for 30 min at 37°C in 5% CO_2_, and the cells were observed by fluorescence microscopy (Axio observer. Z1; Zeiss) at 100× magnification. To stain live cell nuclei, NucBlue Live Cell Stain ReadyProbes reagent, which includes Hoechst 33342 (Life Technologies) was added to each well for 20 min at room temperature.

### Measurement of cellular glutathione content

Intracellular reduced glutathione (GSH) content was analyzed using the NWLSS Glutathione Assay kit (Northwest Life Science Specialties, LLC, Vancouver, WA). Briefly, in 25 cm^2^ tissue culture flasks, H9c2 cells were plated at 2.5 × 10^4^ cells/mL density and grown at 37°C in 5% CO_2_ until 90% confluence. After incubation, the cells were exposed to CYS9, with and without NAC, at 37°C in 5% CO_2_. For experiments with NAC, the cells were pre-treated as described, and then exposed for 2 hours to CYS9 at 37°C in 5% CO_2_ with NAC. Untreated cells were used for control. After treatment, cells were collected and washed twice with ice-cold PBS. The cells were then re-suspended with assay buffer at a density of 1.0 × 10^6^ cells/mL and homogenized by sonication. The lysates were centrifuged at 10,000 rpm for 2 min at 4°C and the supernatant was collected. To remove protein from the supernatant, an equal volume of 5% metaphosphoric acid (MPA) (Sigma) solution was added to 150 μL of supernatant and this mixture was then was vortexed, centrifuged at 10,000 rpm for 2 min at 4°C. This treated sample of supernatant was collected and assayed for GSH content according to the manufacturer’s instructions.

### Measurement of HL-60 cell viability

To determine if NAC, ISO or BIO impair the antitumor efficacy of CY, HL-60 cells were exposed to CYS9 with and without NAC or ISO or BIO. In 24-well plates, HL-60 cells suspended at 4 × 10^5^ cells/mL density in 10% FBS supplemented with RPMI-1640 were exposed to CYS9 at 37°C in 5% CO_2_ for 2 hours; the sample media were then replaced with fresh medium and incubated for a further 6 hours. For experiments with NAC or ISO, the cells were pre-treated as described above in the third paragraph of ‘Analysis of cell viability’, and then exposed for 2 hours to CYS9 at 37°C in 5% CO_2_ with each of the reagents. For experiments with BIO, the cells were co-incubated with CYS9 plus BIO as described above in ‘Analysis of cell viability’. Finally, the CY-exposed samples were evaluated using MTT assays.

### Statistical analysis

Data are presented as mean ± SD. Statistical analysis was performed using StatView version 5.0 for Windows (SAS, Institute Inc., Cary, NC). Treatment effects were established by nonparametric Wilcoxon tests. A probability value of less than 0.05 was considered to be statistically significant.

### Approval of the ethics committee

Ethics approval for this study was obtained from the Ethics Committee of Kagoshima University Hospital. Written informed consent was obtained from the parents of all the children in the study and assent was obtained from all children 10 years or older.

## Results

### Pharmacokinetics of high-dose CY in patients

**Fig 3:**
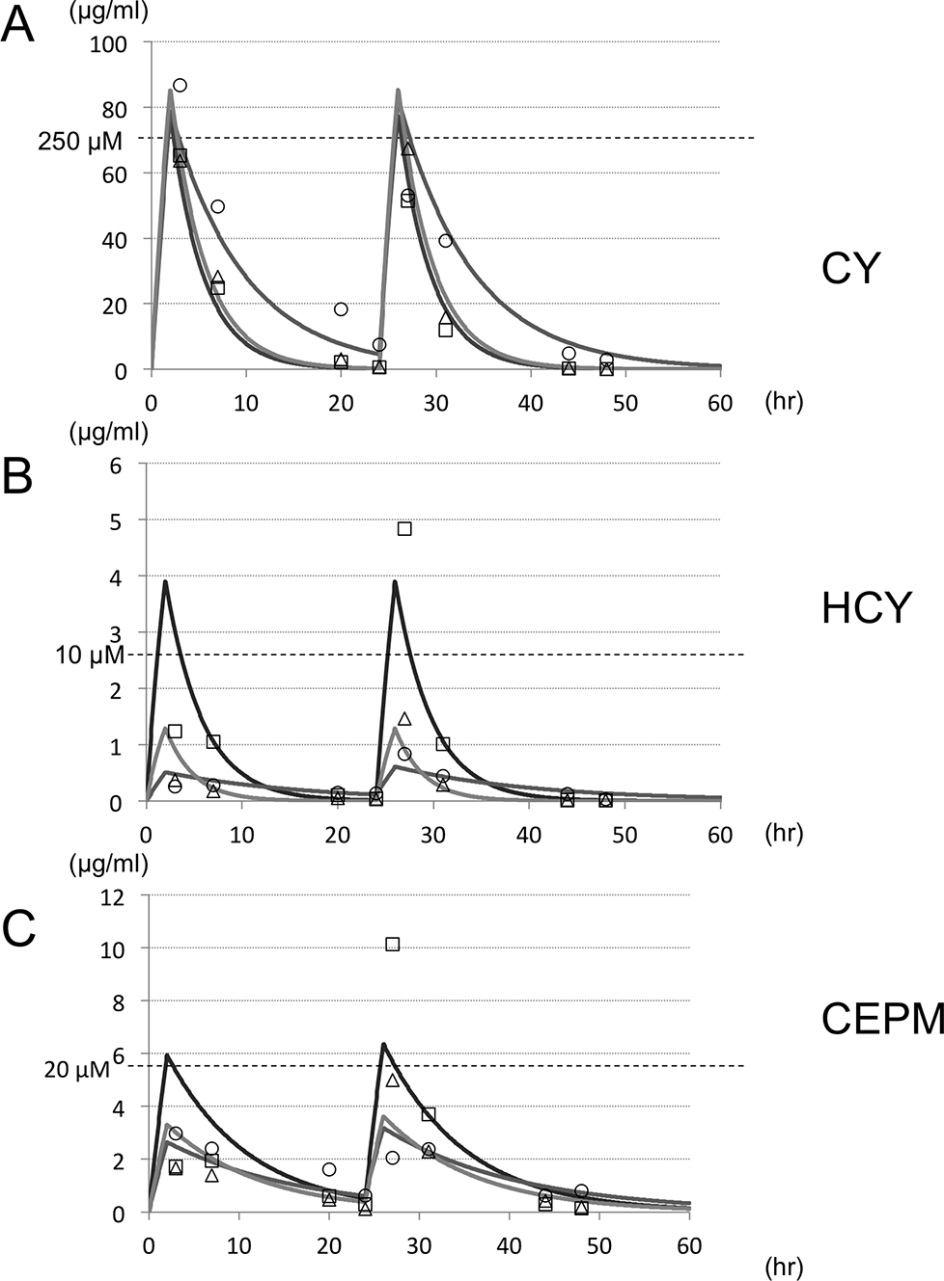
Pharmacokinetics of high-dose cyclophosphamide in patients. We measured CY and CY metabolites in blood plasma samples from patients receiving high-dose CY and here present the concentration–time profiles obtained from all three patients for (A) CY, (B) HCY, and (C) CEPM. Underlying diseases were acute mixed lineage leukemia (male, 8 y.o.; open square) and granulocytic sarcoma (male, 17 y.o.; open circle), and acute myeloid leukemia (male, 1 y.o.; open triangle).

To determine the concentration of CY in this experiment, we measured CY and CY metabolites in blood plasma samples from patients receiving high-dose CY. Fig 3 shows concentration–time profiles obtained from all three patients for CY, HCY, and CEPM. The underlying diseases were acute mixed lineage leukemia (male, 17 y.o.), granulocytic sarcoma (male, 8 y.o.), and acute myeloid leukemia (male, 1 y.o.). The average concentration of CY at 3 hours after administration of high-dose CY therapy was 257 ± 46 μM. Area under the curve (AUC) variability for CY was 2.0×; HCY, 3.8×; CEPM, 1.5×, and HCY/CY 4.9×. No overt cardiac failures nor abnormal ECGs were observed. All three patients underwent engrafting. One patient (acute myeloid leukemia) relapsed after HSCT and died owing to the underlying disease. The other two patients are still alive with no sequelae and have been in continuous remission.

### Myocardial cytotoxicity induced by CY metabolized by S9 mix

**Fig 4:**
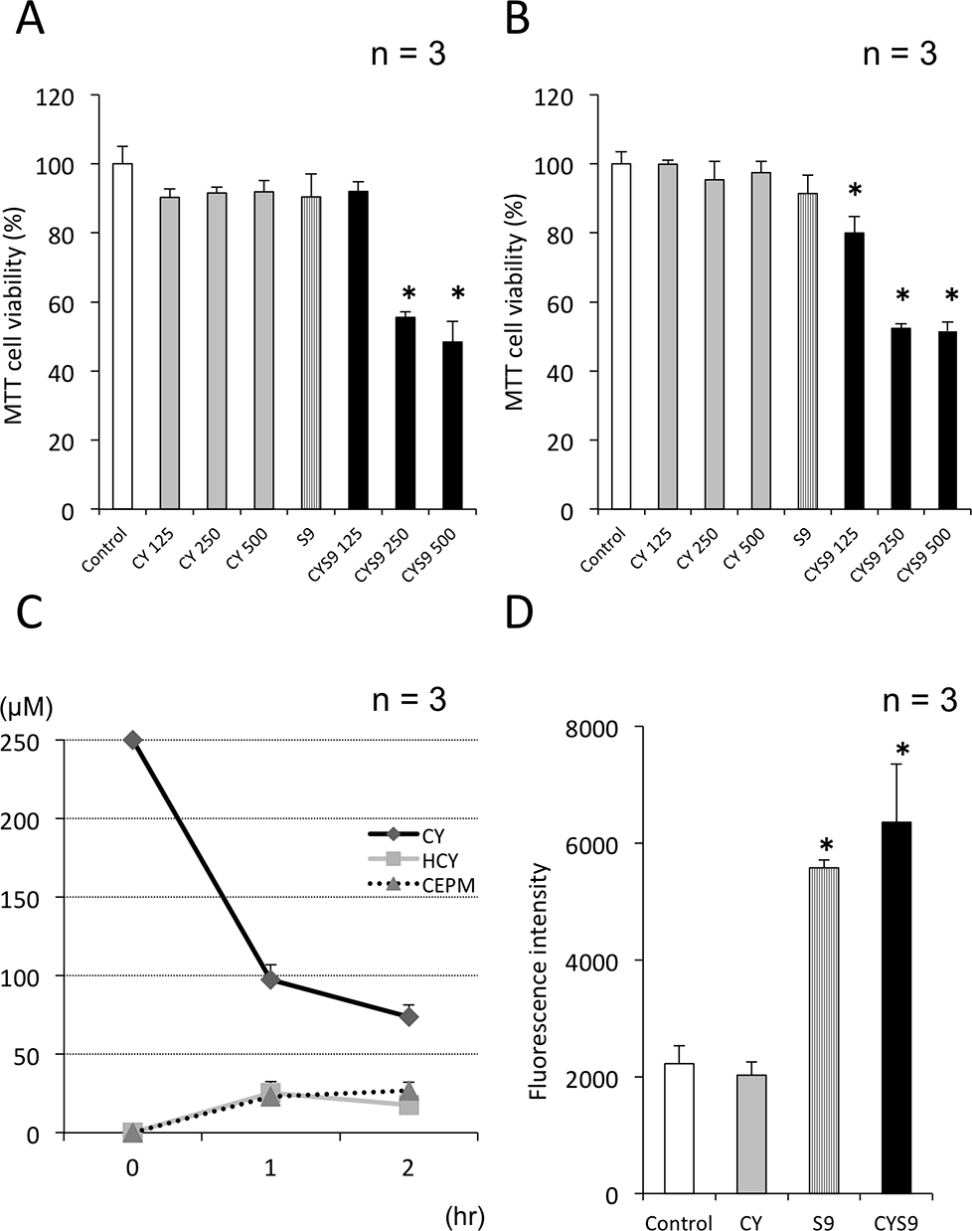
Myocardial cytotoxicity induced by CY metabolized by S9 mix. H9c2 cell viability after (A) 24-hour and (B) 48-hour exposure to CY alone and CY metabolized by S9 fraction of rat liver homogenate mixed with co-factors (CYS9) was assessed by MTT assay (mean + SD from 3 independent experiments). (C) The changes of CY and its metabolites HCY and CEPM concentration in H9c2 cell culture media exposed to CYS9 (mean + SD from 3 independent experiments). (D) Fluorescence intensities, corresponding to levels of H_2_O_2_, in control samples or cells exposed to 250 μM CY, S9, CYS9 for 1 hour (mean + SD from 3 independent experiments). Fluorescence intensity is shown in arbitrary units. *p < 0.05 compared with control.

Assay results for MTT showed that treatment with CY (125–500 μM) or S9 did not induce cytotoxicity. CYS9, however, exhibited myocardial cytotoxicity at 24 hours when CY concentration was 250 μM or more (Fig 4A). At 48 hours, MTT assay results showed that even CY 125 μM CYS9 exhibited myocardial cytotoxicity (Fig 4B). After 250 *μ*M of CY was metabolized in S9 mix for 2 hours, the following metabolite concentrations were detected: CY, 73.6 ± 8.0 μM; HCY, 17.6 ± 4.3 μM; and CEPM 26.6 ± 5.3 μM (Fig 4C). In intracellular ROS, measured fluorescence intensity of cells by using an microplate reader, intracellular levels of H_2_O_2_ in CYS9 (CY 250 μM) samples were higher than in control samples; with S9 on its own, intracellular levels of H_2_O_2_ increased only to the same level as with CYS9 (Fig 4D).

### Inhibition of CYS9-induced cell cytotoxicity by candidate cardioprotectant agents

**Fig 5:**
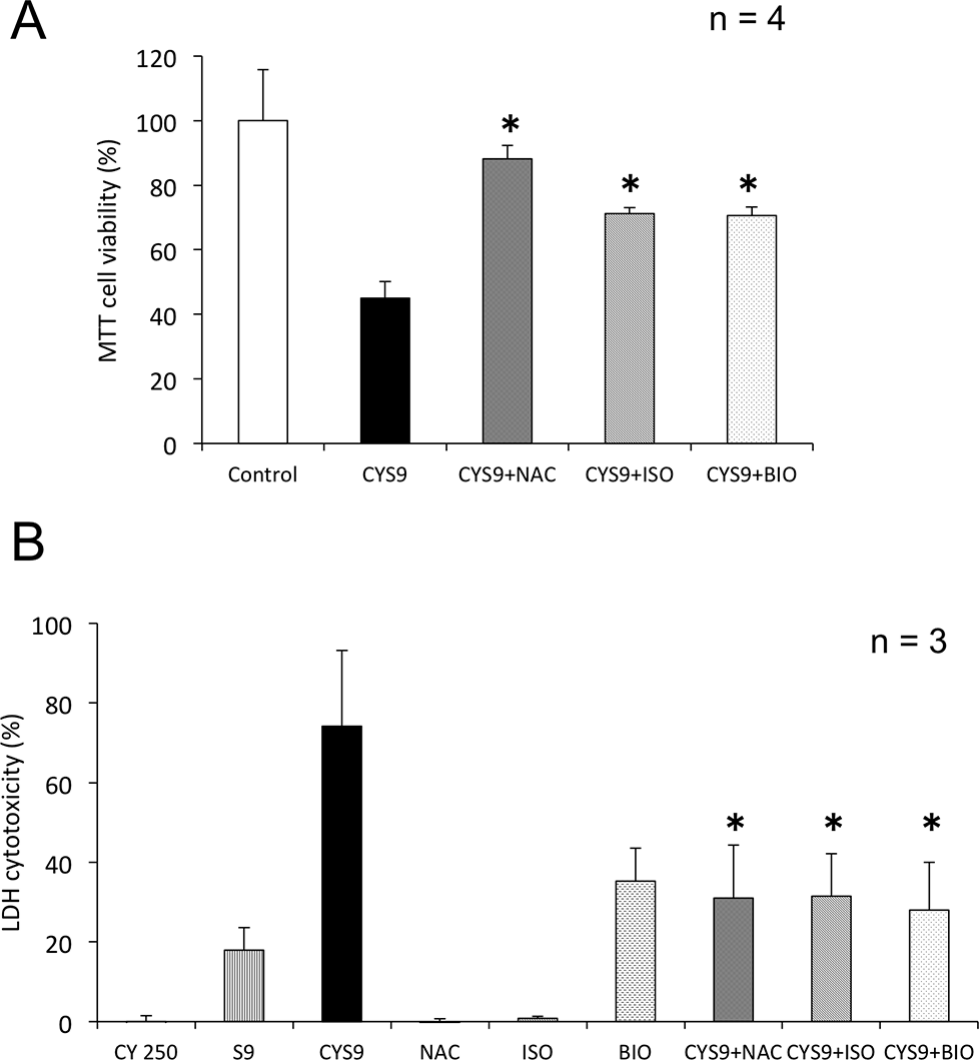
Inhibition of CYS9-induced cell cytotoxicity by candidate cardioprotectant agents. (A) The effect of candidate cardioprotectant agents (NAC, isorhamnetin (ISO), and β-ionone (BIO)) on cytotoxicity of CYS9 in H9c2 cells after 24-hour exposure. (mean + SD from 2 independent experiments conducted in duplicate). *p < 0.05 compared with CYS9 group. (B) The effects of candidate cardioprotectant agents against LDH release from H9c2 cells exposed to CYS9 for 2 hours. (mean + SD from 3 independent experiments). *p < 0.05 compared with CYS9 group.

**Fig 6:**
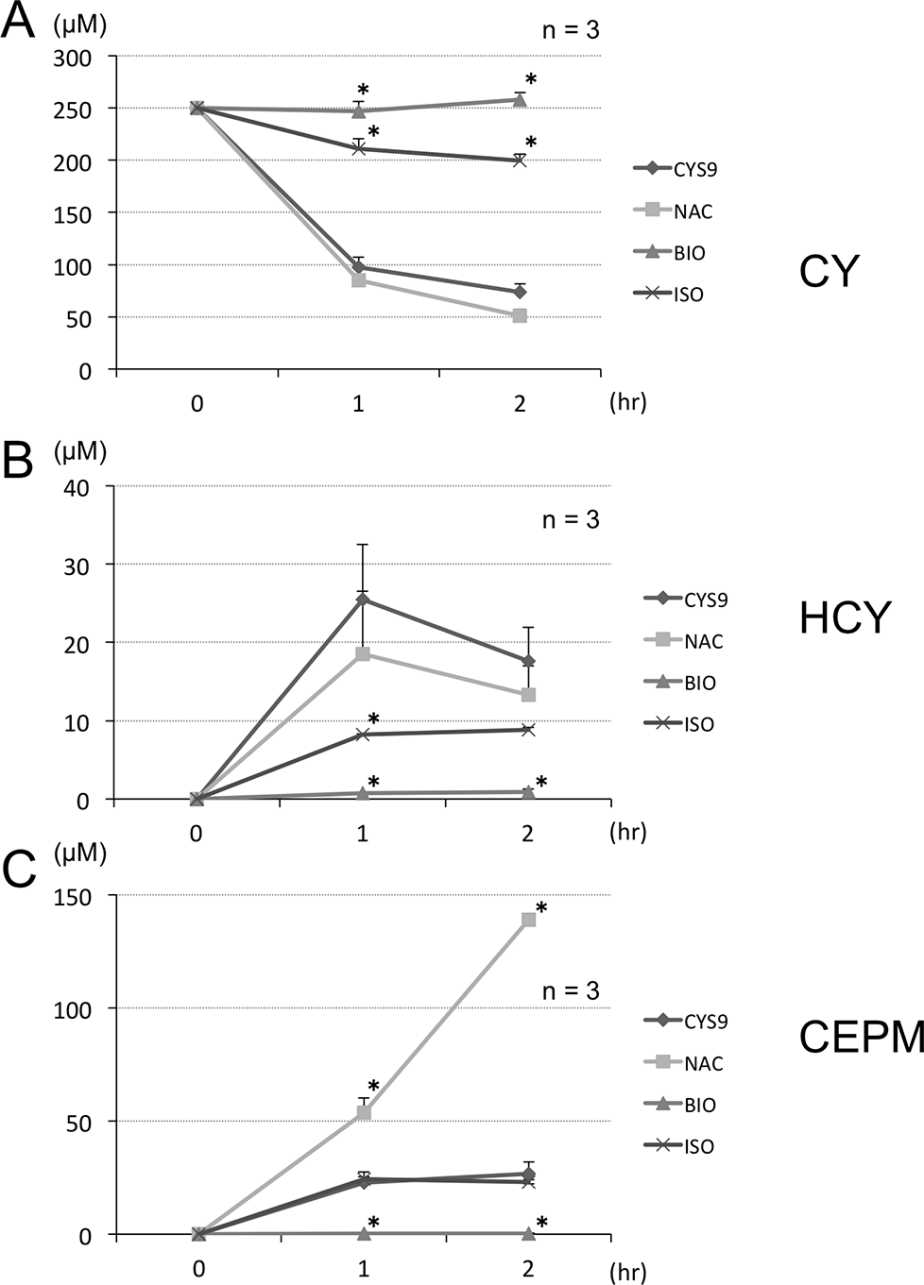
CY and CY metabolites results from LC/MS/MS assays of culture supernatants of CYS9 with and without candidate cardioprotectant agents. H9c2 cells were exposed to CYS9 for 1 or 2 hours with and without candidate cardioprotectant agents (NAC, ISO, and BIO). Changes in concentration in H9c2 cell culture media of (A) CY and its metabolites (B) HCY and (C) CEPM was evaluated using LC/MS/MS. (mean + SD from 3 independent experiments). *p < 0.05 compared with CYS9 group.

**Fig 7:**
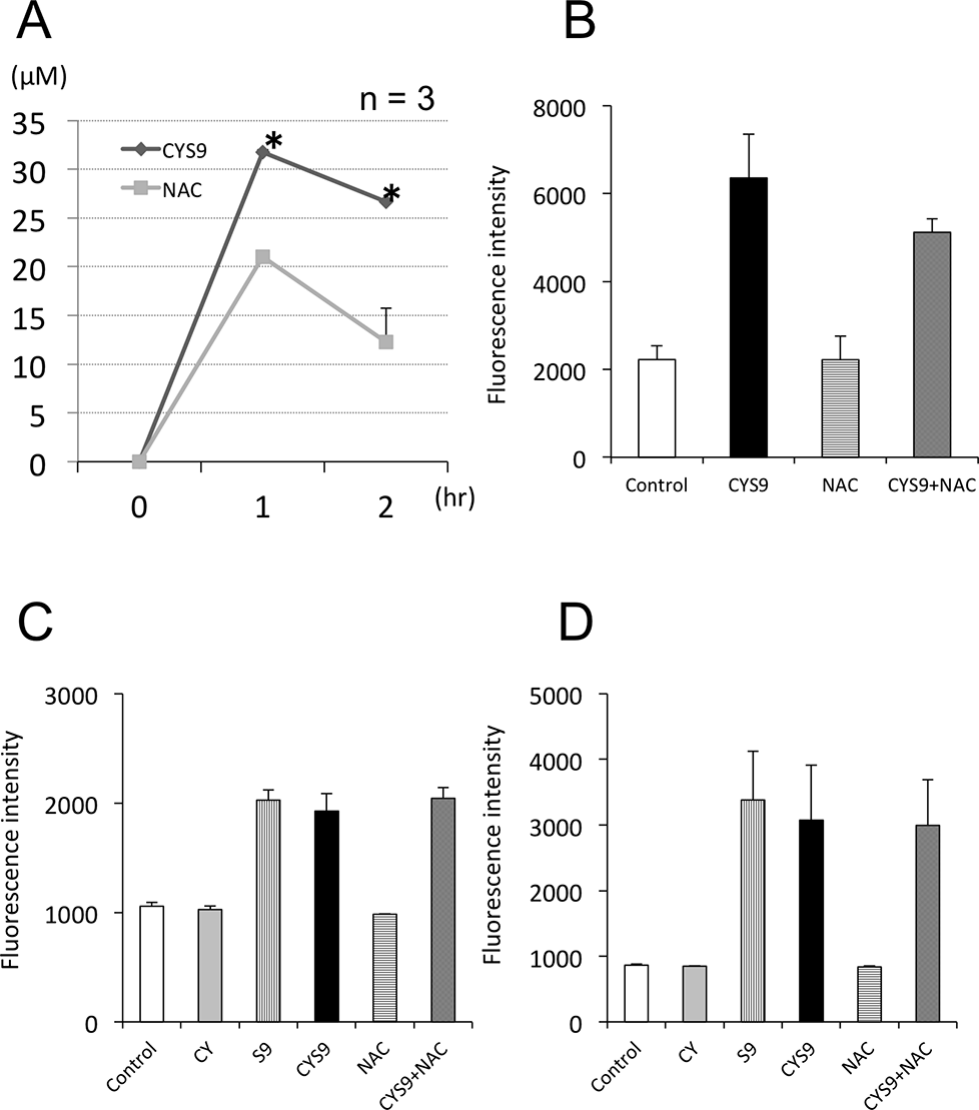
The concentration of acrolein in cell culture media and ROS generation in H9c2 cells after exposure to CYS9 with and without NAC. (A) H9c2 cells were exposed for 1 and 2 hours to CYS9 with and without NAC. The changes of acrolein in culture media was measured using HPLC. (mean + SD from 3 independent experiments). Effect of NAC on ROS generated by CYS9, as shown by fluorescence intensity of (B) DCFH, (C) APF, and (D) HPF in cells exposed for 1 hour to CYS9 or CYS9 plus NAC. Fluorescence intensity is shown in arbitrary units. (mean + SD from 3 independent experiments). *p < 0.05 compared with CYS9 group.

In testing for MTT and release of LDH, assay results showed that NAC, ISO, and BIO all inhibited CYS9-induced cytotoxicity (Fig 5A and 5B). While metabolism of CY was statistically significantly inhibited by treatment with ISO or BIO, pre-treatment with NAC did not result in similar inhibition (Fig 6A–6C). Compared with control results, we observed no difference in HCY, statistically significantly more CEPM (Fig 6B and 6C), and statistically significantly less acrolein (Fig 7A). Furthermore, NAC pre-treatment did not affect intracellular ROS levels, including superoxide, H_2_O_2_, HOCl and the •OH, produced by CYS9 (Fig 7B–7D).

### Optical and fluorescence images of H9c2 cells exposed to CY, CYS9, and CYS9 plus NAC

**Fig 8:**
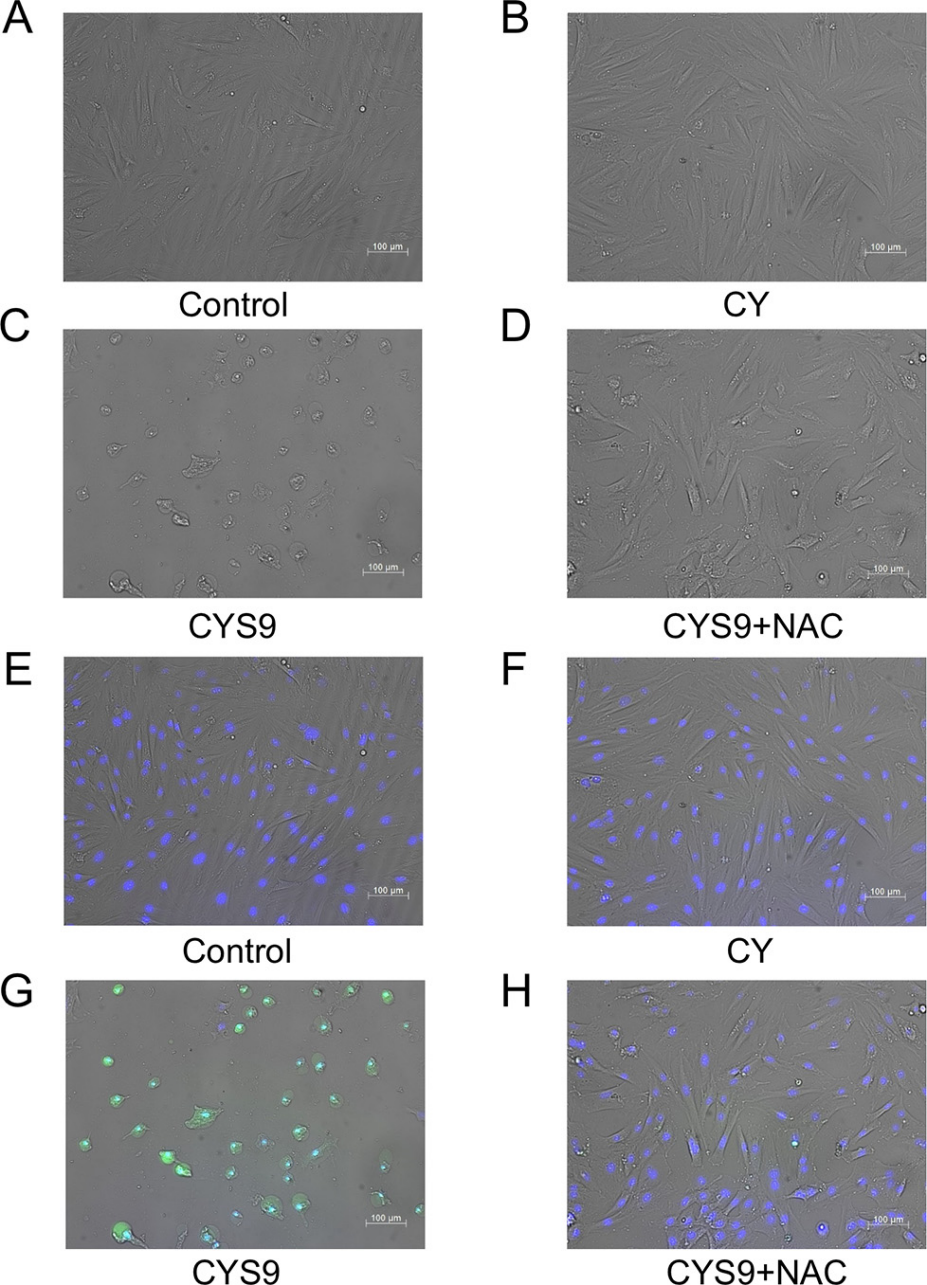
Optical and fluorescence images of H9c2 cells exposed to CY, CYS9, and CYS9 plus NAC. A, B, C, D: Optical images at 24-hour exposure. (A) Control (unexposed H9c2 cells), (B) H9c2 cells exposed to 250 μM CY, (C) H9c2 cells exposed to CYS9, and (D) H9c2 cells exposed to CYS9 presence of 1 mM NAC. Magnification, 100×. Bar = 200 μm. E, F, G, H: Induction of apoptosis in H9c2 cells by CYS9 with or without NAC. Living cell nucleii stained by Hoechst 33342 are blue. Apoptotic cells stained by FITC-conjugated probes are green. (E) Control (unexposed H9c2 cells), (F) H9c2 cells exposed for 2 hours to 250 μM CY, (G) H9c2 cells exposed to CYS9—green dots indicate apoptotic cells. (H) H9c2 cells exposed to CYS9 with 1 mM of NAC. Magnification, 100×. Bar = 200 μm.

Live-cell imaging also confirmed acute cytotoxicity in CYS9 samples, which was inhibited by NAC (Fig 8A–8D). In CYS9 samples without NAC, cells appeared either shrunken or irregularly shaped; but with NAC, the cells had a very similar appearance to those in control samples. The protective effect of NAC on H9c2 cells was corroborated using Hoechst 33342 staining and fluorescent assays of Caspase-3/7 activity (Fig 8E–8H). In Fig 8H, with round-shaped nuclei and homogeneous blue fluorescence intensity, the cells in CYS9 samples treated with NAC have a similar appearance to normal cells. As Fig 8G shows, caspase-3 and caspase-7 activity was greater in CYS9 (CY 250 μM) samples; such activity was suppressed in samples pre-treated for 2 hours with 1 mM NAC (Fig 8H).

### GSH involvement in NAC protection of H9c2 cells

**Fig 9:**
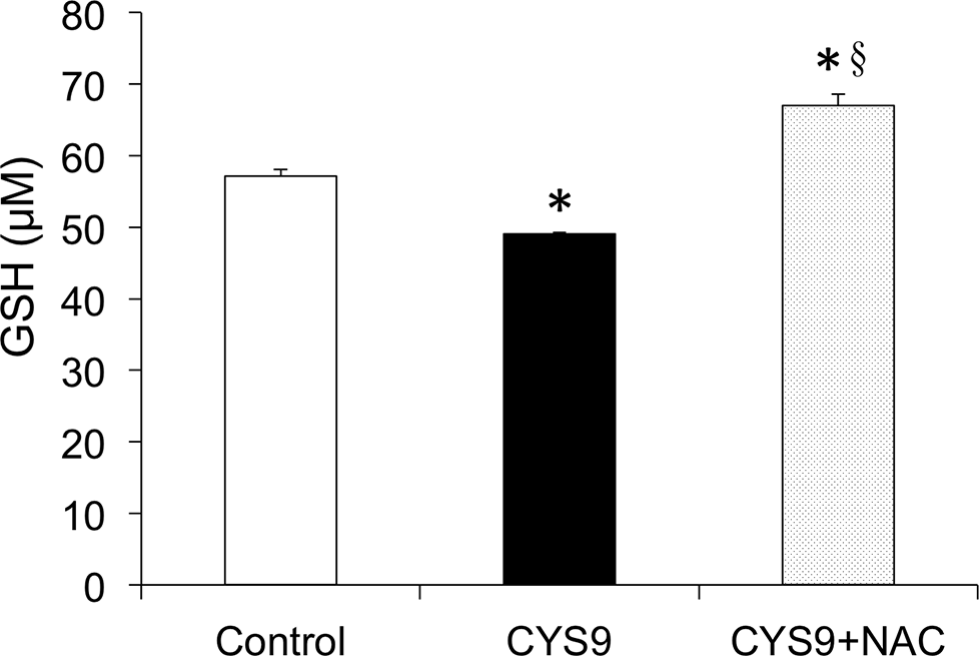
Reduced glutathione levels in H9c2 cells after treatment with CYS9 in presence or absence of NAC. Effects of NAC on reduced glutathione (GSH) levels in H9c2 cells exposed to CYS9 for 2 hours. (mean + SD from 3 independent experiments). *p < 0.01 compared with control group; §*p* < 0.01 compared with CYS9 group.

CYS9 was statistically significantly (*p* < 0.01) associated with decreased cardiomyocyte GSH. In myocytes pretreated with NAC, CYS9 did not significantly diminish cellular GSH (Fig 9). Interestingly, the GSH in the CYS9 with NAC group was statistically significantly (*p* < 0.01) higher than in the control group, suggesting that NAC provides cells with additional cysteine for GSH synthesis (Fig 9).

### Acrolein-induced cytotoxicity in H9c2 cells with or without NAC

**Fig 10:**
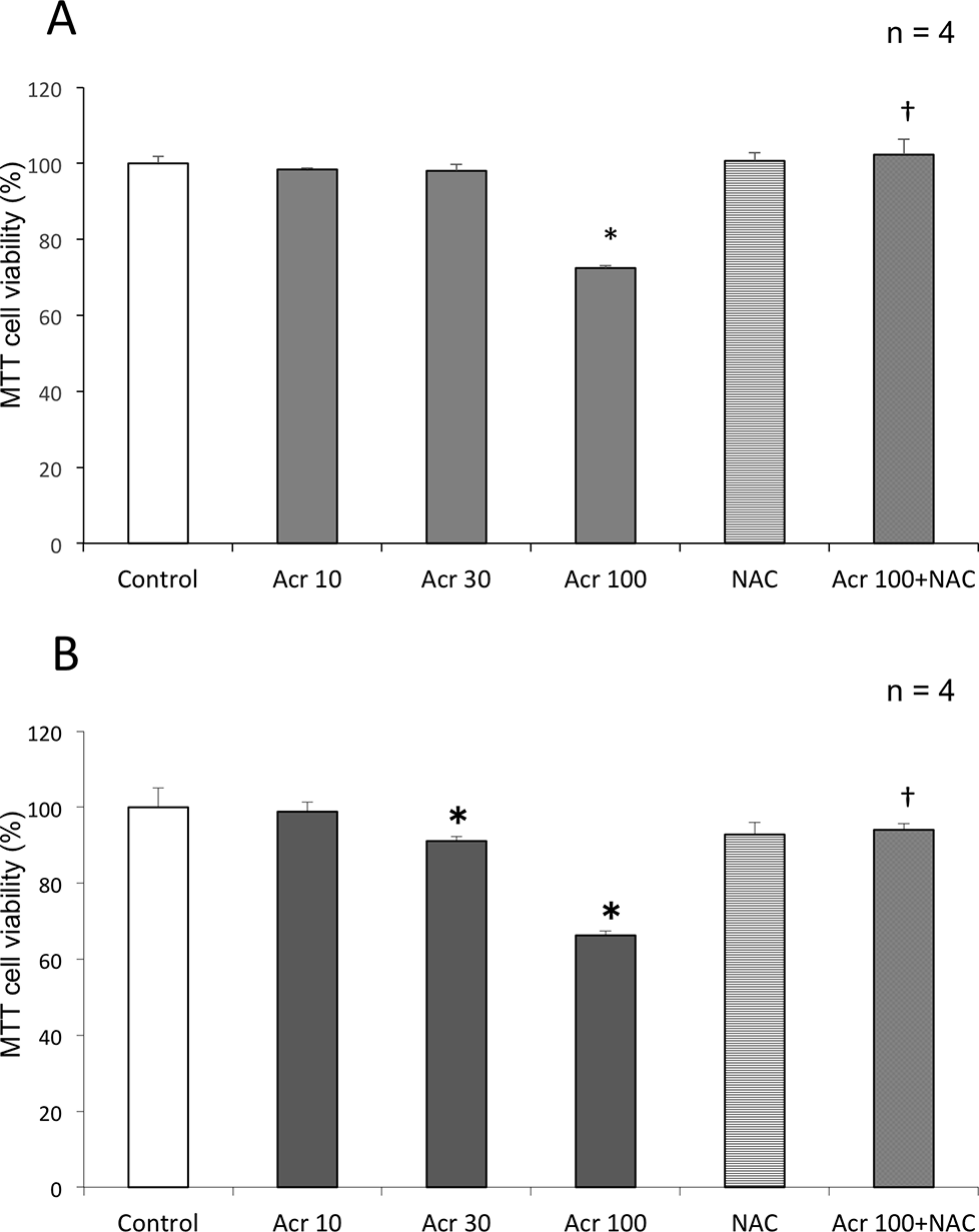
Myocardial cytotoxicity induced by acrolein. H9c2 cell viability after (A) 24-hour and (B) 48-hour exposure to acrolein (Acr) with or without NAC was assessed by MTT assay (mean + SD from 2 independent experiments conducted in duplicate). *p < 0.05 compared with control group. †*p* < 0.05 compared with acrolein 100 μM group.

H9c2 cells were treated with 0, 10, 30, 100 μM acrolein for 24 hours or 48 hours, and the viability of the cells was measured by MTT assay. Treatment with 10 μM acrolein did not affect H9c2 cell viability, but higher concentrations statistically significantly (*p* < 0.05) decreased cell viability, indicating that the cytotoxicity of acrolein in H9c2 cells was concentration dependent (Fig 10).

### CYS9-induced cytotoxicity in HL-60 cells with candidate cardioprotectant agents

**Fig 11:**
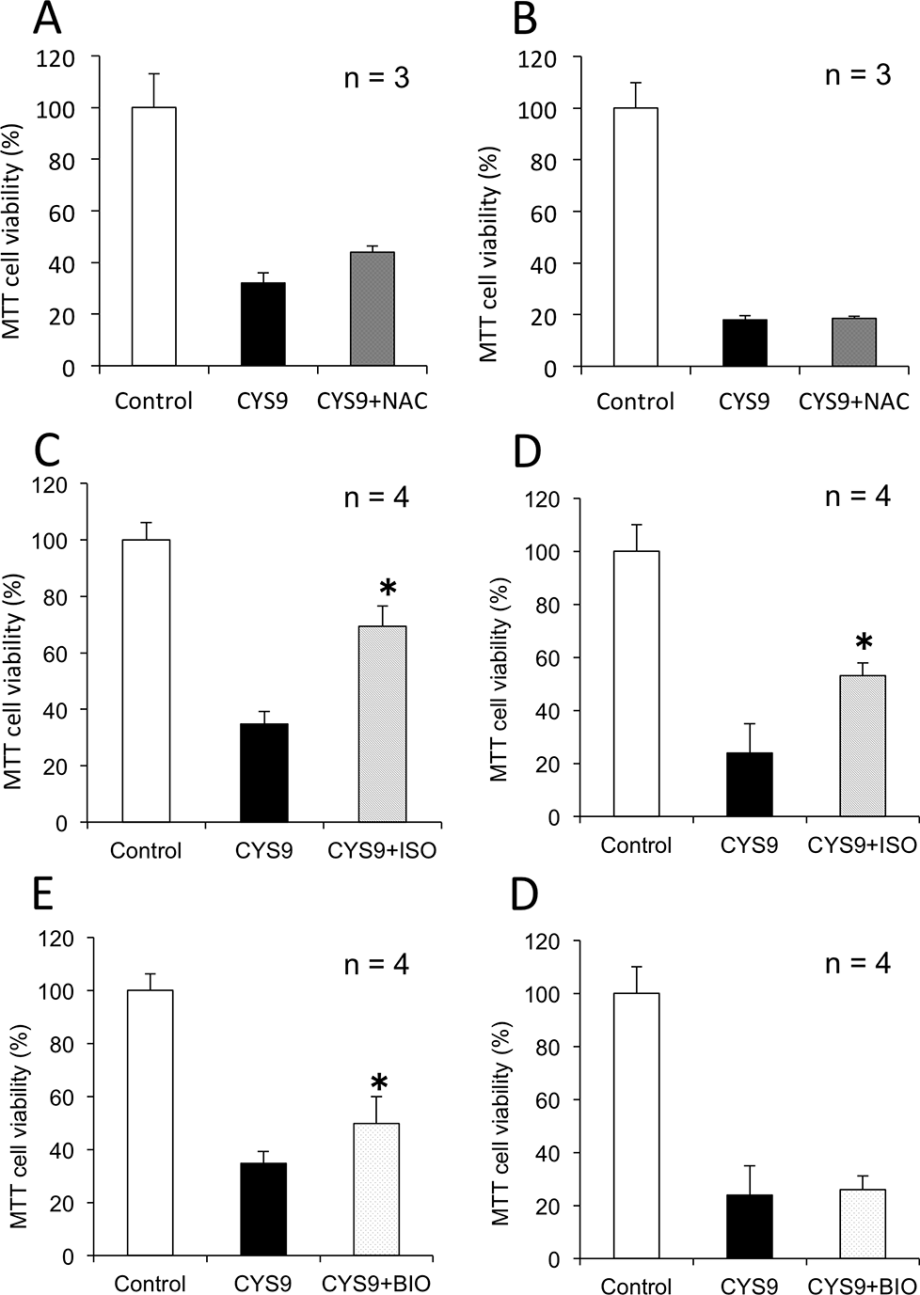
CYS9-induced cytotoxicity in HL-60 cells with candidate cardioprotectant agents. Cell viability was assessed by MTT assay after HL-60 cells were exposed to CYS9 with and without NAC or ISO or BIO: results show cell viability (mean + SD from 3 or 4 independent experiments) after exposure to CYS9 for 24 hours (A, C, E) or 48 hours (B, D, F). *p < 0.05 compared with CYS9 group.

To determine whether NAC or ISO or BIO affected the antitumor activity of CYS9, tests were carried out on cells from cancer cell line HL-60. As Fig 11 shows, CYS9 pre-treated with NAC produced as great a decrease in cancer cell viability as CYS9 used alone. However, CYS9 pre-treated with ISO for 24 hours and 48 hours or co-incubated with BIO for 24 hours statistically significantly (*p* < 0.05) inhibited the antitumor activity of CYS9. This finding confirms that of the three candidates, only NAC protects against the cardiotoxicity of CYS9 while not inhibiting its antitumor activity.

## Discussion

Cardiotoxicity is the dose-limiting toxic effect of CY, and is observed only after administration of high doses (60 mg/kg daily × 2 days or 50 mg/kg daily × 4 days) [1, 18, 19]. The most severe clinical manifestation is hemorrhagic necrotic perimyocarditis. Cellular mechanisms of CY-induced cardiotoxicity have been thought to be mediated by an increase in free oxygen radicals [20–23]. Results from the present study enable construction of a novel hypothesis that the cardiotoxic action of high-dose CY may be due to the formation of acrolein, a metabolite of CY.

Our three hematological cancer patients showed pronounced pharmacokinetic variability in CY, HCY and CEPM, the highest (4.9-fold) variability being observed in the AUC ratio for HCY/CY. Similar variability has also been reported for other patient groups [24, 25] and may be related to individual susceptibility to side effects, including cardiotoxicity.

CY itself did not have cardiotoxic effects. In *in vitro* bioassays, S9 microsomal fraction of rat liver homogenate is widely used in to evaluate the toxicity of reactive metabolites [26, 27]. Since S9 contains enzymes of the CYP superfamily, phase I metabolism in the liver is partially replicable in the presence of co-factors, such as NADP+, which is used as an electron donor. When CY was metabolized *in vitro* by S9 mix, we ascertained from LC/MS/MS and HPLC results that it would produce similar levels of metabolic substance concentrations as in patients who receive high-dose CY (Figs 3 and 4C). At concentrations of 250 μM CY or more, CYS9 exhibited myocardial cytotoxicity at 24 hours (Fig 4A). This data corresponds with findings that CY cardiotoxicity is only observed after high-dose administration, but not with low-doses (1–3 mg/kg) or intermediate-doses (15–40 mg/kg). Several ROS, including O_2_•−, H_2_O_2_, HOCl, and •OH, were generated by high-dose (CY 250 μM) exposure to CYS9. The intracellular levels of H_2_O_2_ found with S9 on its own, however, were similar to the levels found with CYS9 (Figs 4D, 7C and 7D). It seems that some of the cytotoxic effects of the S9 mix may be due to toxicants resulting from, in the presence of S9, the peroxidation of microsomal lipids [28]. On its own, S9 copiously generates ROS, as a result it may be hard to detect lesser ROS production by CY metabolites. Other researchers have reported, however, that CY metabolite acrolein increases levels of intracellular ROS [29–31].

In the present study, treatment with CYS9 (CY 250 μM) was shown to initiate the activation of caspase-3 and caspase-7 in H9c2 cardiomyocytes (Fig 8G). Since caspase-3 and caspase-7 are the key executioners of apoptosis, CYS9 induced apoptotic death.

We considered three agents, NAC, ISO and BIO, as a candidate cardioprotectant agents. NAC is widely used as a mucolytic agent or an antidote for acetaminophen overdose hepatotoxicity. As an antioxidant containing thiol groups, NAC is a form of amino-acid cysteine that stimulates glutathione synthesis and scavenges free radicals [32]. Furthermore, NAC is a powerful scavenger of acrolein [33]. ISO is an antioxidant found in medicinal plants which are frequently used for the prevention and treatment of cardiovascular diseases [34]. A recent report suggested that ISO may be useful in adjuvant therapy during long-term clinical use of doxorubicin [11]. BIO is a cyclic terpenoid (C_13_) compound found in a variety of edible and aromatic plants. It has been demonstrated that BIO is a potent inhibitor of CYP2B isoenzymes in rat liver microsomes [12].

When H9c2 cells were treated with NAC, ISO, and BIO, damage caused by exposure to CYS9 was inhibited (Fig 5). ISO and BIO inhibited metabolism of CY itself (Fig 6A–6C). This result suggests that cardiac cell damage may depend on individual differences in CY metabolic capacity. As preventative drugs, however, both BIO and ISO seem likely to interfere with the antitumor effects of CY and are thought to be unsuitable for supplementing CY therapy.

In our study, the results of four experiments provided firm evidence that NAC protects against CYS9-induced apoptosis and cell death: MTT assay, LDH release, Hoechst33342 staining and fluorescent assays of Caspase-3/7 activity. More promisingly, pre-treatment with NAC did not inhibit CY metabolism: compared to control samples, while HCY concentration was similar, CEPM concentration statistically significantly increased (Fig 6A–6C). Less acrolein, however, was present after exposure to NAC (Fig 7A). Furthermore, the cytotoxicity of acrolein was concentration dependent in H9c2 cells (Fig 10). This result is consistent with other reports showing that acrolein, as a reactive aldehyde, is a major component of environmental pollutants and is implicated in cardiac diseases [35–38]. We speculate that NAC inhibited CYS9-induced cardiotoxicity by directly scavenging acrolein [33]. This acrolein would otherwise inhibit ALDH1 activity [39]; consequently, pre-treatment with NAC results in statistically significantly greater CEPM concentration. Cardiac myocytes may defend against the cytotoxicity of high-dose CY by expressing ALDH1, which plays a similar defensive role in donor regulatory T cells in PTCy [40], in cancer stem cells [41], and in hematopoietic stem cells [42].

Pre-treatment with NAC did not affect the total amount of intracellular ROS produced by CYS9 (Fig 7B–7D). NAC, which is known to be a free radical scavenger, did not inhibit these increased ROS levels. It is possible that any ROS inhibition by NAC may not have been detectable, however, masked by the copious amounts of that S9 independently generates. So far, cellular mechanisms of CY-induced cardiotoxicity have been attributed to an increase in free oxygen radicals and induced apoptosis [23, 43]. GSH is a potent intracellular anti-oxidant and it has been reported that intracellular depletion of GSH enhances cytotoxicity and apoptosis [44]. Fig 9 shows that while CYS9 significantly decreased the intracellular level of GSH, the presence of NAC increased intracellular GSH. This NAC-increased GSH level may an important factor in the mechanism for protecting H9c2 cells.

Since acrolein seems to be heavily implicated in the onset of cardiotoxicity, any competitive metabolic processing of AldoCY that reduces its transformation to acrolein is likely to be an important mechanism for preventing cardiotoxicity. In ongoing clinical research, we intend to measure CY and CY metabolites, including acrolein, in patients who receive the high-dose CY. Since NAC inhibited cytotoxicity induced by CY plus S9 mix, we think it might serve as a novel cardioprotective agent to prevent the occurrence of high-dose CY-induced cardiotoxicity. Since NAC is already being used in HSCT settings for preventing oral mucositis from day one of high-dose chemotherapy including CY [45], clinical application of NAC to prevent high-dose CY cardiotoxicity is feasible.

## Conclusions

In summary, our findings provide evidence that the onset of myocardial damage triggered by high-dose CY is likely to be determined by an individual patient’s metabolic amounts of HCY and AldoCY and the degree of ALDH1 activation in their myocardial cells. Consequently, through a mechanism related to its ability to inhibit acrolein, NAC may attenuate cardiotoxicity associated with high-dose CY.
